# Increased Peptide Contacts Govern High Affinity Binding of a Modified TCR Whilst Maintaining a Native pMHC Docking Mode

**DOI:** 10.3389/fimmu.2013.00168

**Published:** 2013-06-26

**Authors:** David K. Cole, Malkit Sami, Daniel R. Scott, Pierre J. Rizkallah, Oleg Y. Borbulevych, Penio T. Todorov, Ruth K. Moysey, Bent K. Jakobsen, Jonathan M. Boulter, Brian M. Baker, Yi Li

**Affiliations:** ^1^Cardiff University School of Medicine, Heath Park, Cardiff, UK; ^2^Immunocore Limited, Oxon, UK; ^3^Department of Chemistry and Biochemistry, University of Notre Dame, Notre Dame, IN, USA; ^4^Oxford Nanopore Technologies Ltd., Oxford, UK; ^5^Center for Infection and Immunity, Guangzhou Institutes of Biomedicine and Health, Chinese Academy of Sciences, Guangzhou, China

**Keywords:** human T leukocyte virus type 1, crystal structure, peptide-major histocompatibility complex, surface plasmon resonance, T cell, T cell receptor, A6 TCR, high affinity TCR

## Abstract

Natural T cell receptors (TCRs) generally bind to their cognate pMHC molecules with weak affinity and fast kinetics, limiting their use as therapeutic agents. Using phage display, we have engineered a high affinity version of the A6 wild-type TCR (A6wt), specific for the human leukocyte antigen (HLA-A^∗^0201) complexed with human T cell lymphotropic virus type 1_11–19_ peptide (A2-Tax). Mutations in just 4 residues in the CDR3β loop region of the A6wt TCR were selected that improved binding to A2-Tax by nearly 1000-fold. Biophysical measurements of this mutant TCR (A6c134) demonstrated that the enhanced binding was derived through favorable enthalpy and a slower off-rate. The structure of the free A6c134 TCR and the A6c134/A2-Tax complex revealed a native binding mode, similar to the A6wt/A2-Tax complex. However, concordant with the more favorable binding enthalpy, the A6c134 TCR made increased contacts with the Tax peptide compared with the A6wt/A2-Tax complex, demonstrating a peptide-focused mechanism for the enhanced affinity that directly involved the mutated residues in the A6c134 TCR CDR3β loop. This peptide-focused enhanced TCR binding may represent an important approach for developing antigen specific high affinity TCR reagents for use in T cell based therapies.

## Introduction

CD8^+^ αβ T cells recognize mainly intracellularly expressed antigens through an interaction mediated by the cell surface expressed T cell receptor (TCR). Intracellular proteins, processed by the proteasome into short peptides (generally 8–13 amino acids in length), are presented to CD8^+^ T cells on the surface of almost all nucleated human cells by class I major histocompatibility complex proteins (pMHCI). TCR recognition of pMHCI initiates CD8^+^ T cell activation and the adaptive immune response. The ability of CD8^+^ T cells to scrutinize the intracellular environment provides an important mechanism to target aberrant disease epitopes that would be otherwise hidden from the immune system. Thus, CD8^+^ T cells play an important role during viral infections (Miles et al., 2010), which typically elicit strong CD8^+^ T cell responses, many of which have been well-characterized, including those to HTLV-1 (Bieganowska et al., 1999; Vine et al., 2004), although some viruses can escape CD8^+^ T cell mediated clearance (Klenerman and Zinkernagel, 1998; Overbaugh and Bangham, 2001). Cancers too, as a result of their malignant transformation, have altered protein expression causing the presentation of tumor-associated peptide antigens (TAPAs) (Renkvist et al., 2001; Cole et al., 2009). However, although these TAPAs can elicit a host CD8^+^ T cell response, this is often insufficient to cause tumor rejection (Blohm et al., 2002; Parkhurst et al., 2004). CD8^+^ T cell responses are also integral to the initiation and progression of many autoimmune diseases, possibly through the unwanted recognition of self-peptide antigens (Bulek et al., 2012). The TCR/pMHC interaction, which governs CD8^+^ T cell responses, is therefore an attractive therapeutic target in many varied diseases, particularly in cases where a disease-associated peptide antigen “target” has been unambiguously established.

However, unlike antibodies that can undergo somatic hypermutation and bind with high affinity (*K*_D_ = nM–pM) and long half-lives (typically hours), TCRs are selected in the thymus to bind with weak affinity (*K*_D_ = 100 nM–270 μM) and short half-lives (typically seconds) (Cole et al., 2007; Bridgeman et al., 2012). Why TCRs are selected to bind within this weak affinity range is not fully understood, but may represent a balance between self-tolerance and a requirement for T cell cross-reactivity (Mason, 1998; Sewell, 2012; Wooldridge et al., 2012). However, the weak affinity and short half-lives of natural TCR/pMHC interactions imposes limitations on the use of TCRs for targeting cell surface expressed pMHCs, primarily because the short half-life is not adequate for the delivery of therapeutic interventions to target antigens. In order to overcome this limitation, we have recently implemented phage display to generate TCRs with an enhanced affinity for cognate antigen (Li et al., 2005; Dunn et al., 2006; Sami et al., 2007; Varela-Rohena et al., 2008; Liddy et al., 2012) that can be used to target cell surface expressed MHC molecules displaying any disease epitope of interest. Using this technique, we have generated several high affinity TCRs derived from the αβ TCR A6 (A6wt) that are specific for the HTLV-1_11–19_ peptide, presented by HLA-A^∗^0201 (A2-Tax) (Li et al., 2005). High affinity TCRs generated using this method can be used for two distinct type of therapies. The first involves genetically reprograming host T cells so that they express a modified TCR (adoptive therapy) (Morgan et al., 2006; Varela-Rohena et al., 2008). The second involves using a soluble high affinity TCR to deliver a therapeutic payload intravenously (soluble therapy) (Liddy et al., 2012). The optimal TCR affinity for these two types of therapy, in terms for retaining specificity and reactivity, will probably be distinct and will likely be lower for adoptive therapy because of the polyvalent nature of T cell antigen recognition at the cell surface versus a soluble therapy in which the TCR reagent will likely require a longer half-life to effectively target intended disease markers. Understanding how these reagents function at the molecular level is key to determining these parameters and optimizing these types of T cell directed therapies.

Previous structural investigations of TCR/pMHC interactions have shown that TCRs bind with a relatively conserved diagonal orientation (Garboczi et al., 1996; Rudolph et al., 2006), with the α-chain focused toward the N-terminus of the peptide and the β-chain toward the C-terminus. Although exceptions occur (Burrows et al., 2010), this orientation enables the TCR complementarity determining region (CDR)2 loops to be positioned over mainly the MHC surface, the CDR3 loops to be positioned primarily over the peptide and the CDR1 loops positioned in between. This binding mode, and the low native TCR binding affinity, is presumably important for maintaining T cell specificity and antigen sensitivity, and is possibly important in T cell signaling (Adams et al., 2011). However, we have previously shown that just a small number of mutations in the TCR CDR loops can improve the low natural TCR/pMHC binding affinity dramatically (Li et al., 2005; Dunn et al., 2006; Hawse et al., 2012). Thus, it is likely that high affinity TCRs are generated in the thymus, but they are not selected for release into the periphery. In order to better understand the consequences of high affinity TCR interactions, and to provide further insight into: (1) how high affinity TCR binding is mediated and (2) what effects this binding is likely to have on TCR specificity, we solved the atomic structures of a high affinity TCR, A6c134, free, and in complex with A2-Tax. By comparing this structure with the previously published structures for the A6wt TCR (Garboczi et al., 1996; Scott et al., 2011), we provide a molecular explanation for the improved binding of this high affinity TCR.

## Results

### Deconstruction of high affinity A6 TCR variants specific for A2-Tax

In order to generate a high affinity version of the A6wt TCR, we implemented phage display as previously described (Li et al., 2005). This process generated a number of high affinity TCRs, including the mutant A6c134 that varied from the A6wt TCR parental sequence at only four codons, all located within the CDR3β loop (Table 1). It has been previously determined that the A6wt TCR binds to A2-Tax with an affinity of ∼1–3 μM and an off-rate (*t*_1/2_) of ∼7–10 s (Davis-Harrison et al., 2005; Armstrong and Baker, 2007; Cole et al., 2007). In contrast, the engineered high affinity A6c134 TCR bound to A2-Tax with an affinity of 4 nM (nearly 1000 times greater than the A6wt TCR, or ΔΔG° = −3.96 kcal/mol) and an off-rate (*t*_1/2_) of 3900 s (>400 times longer than the A6wt TCR), as determined by surface plasmon resonance (SPR) (Table 1). A6c134 did not bind to other HLA-A2 restricted peptides that were used as negative controls in different SPR experiments, including as A2-ILAKFLHWL, A2-ELAGIGILTV, and A2-YLEPGPVTA demonstrating that A6c134 retained peptide specificity (data not shown). In order to investigate the molecular basis for how this high affinity was generated, we used reverse engineering to construct a range of A6wt-based TCRs containing different combinations of amino acids from the A6c134 CDR3β sequence and, conversely, a range of A6c134-based TCRs containing different combinations of amino acids from the A6wt CDR3β sequence (Table 1). All of these TCRs exhibited very similar on-rates, but showed marked differences in their off-rates (Table 1). In general, the mutations appeared to act cooperatively to enhance affinity. The mutation of the A6wt TCR from R_102_ to Q_102_ had only a small effect upon binding affinity and may have been selected because of the lower toxicity of the TCR to the TG1 phage host (Li et al., 2005).

**Table 1:** **Biophysical analysis of different combinations of “knock-in” and “knock-out” mutations (in bold type and underlined) of A6wt and A6c134 TCRs binding to A2-Tax**.

Mutant	CDR3β	*K*_D_	*k*_on_ (s^−1^ M^−1^)	*k*_off_ (s^−1^)	*t*_1/2_ (s)	ΔG° (kcal/mol)	ΔΔG° (kcal/mol)
A6wt	AGGR	3.2 μM	2.3 × 10^4^	7.4 × 10^−2^	9.3	−7.49	n/a
A6c134M	**M**GGR	1.9 μM	1.8 × 10^4^	3.5 × 10^−2^	20	−7.80	−0.31
A6c134S	A**S**GR	1.8 μM	2.3 × 10^4^	4.1 × 10^−2^	17	−7.83	−0.34
A6c134AE	A**SAE**	9.4 nM	2.3 × 10^4^	5.2 × 10^−4^	1320	−10.94	−3.45
A6c134E	**MSAE**	4.4 nM	5.5 × 10^4^	2.2 × 10^−4^	3120	−11.39	−3.90
A6c134R	**MSA**R	8 nM	1.9 × 10^4^	1.5 × 10^−4^	4500	−11.04	−3.55
A6c134	**MSAQ**	4 nM	4.5 × 10^4^	1.8 × 10^−4^	3900	−11.45	−3.96

*Off-rates were determined by least squares fitting of single-component exponential decay equation to the decay curve following TCR binding to A2-Tax*.

*Half-lives were calculated using the equation *t*_1/2_ = ln2/*k*_off_*.

*ΔΔG° = ΔG° of high affinity A6c134 variant binding to A2-Tax −ΔG° of A6wt binding to A2-Tax*.

### A6c134 TCR bound to A2-Tax using a similar conformation to the A6wt TCR

**Figure 1:**
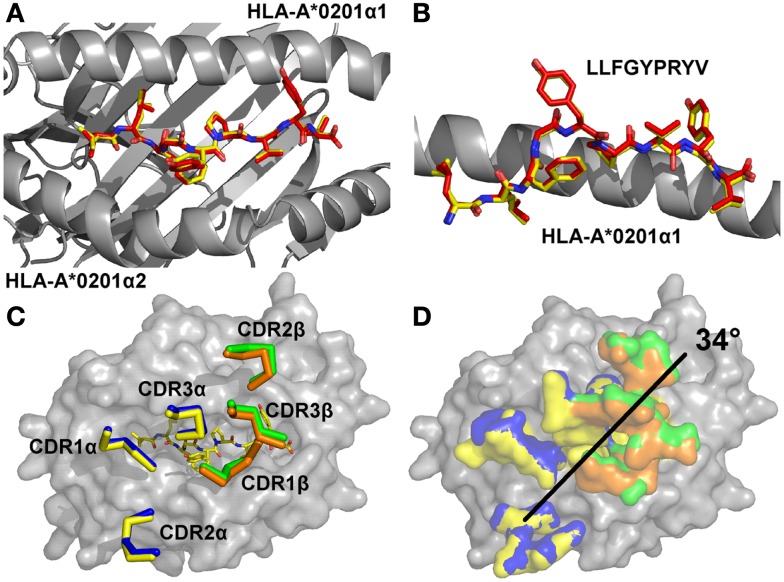
**The overall conformations of A6wt and A6c134 TCRs in complex with A2-Tax are similar**. **(A)** Comparison of peptide conformation in the A6wt TCR (red sticks) and A6c134 TCR (yellow sticks) complex structures looking down on top of the MHC groove (gray sticks). **(B)** Comparison of peptide conformation in the A6wt TCR (red sticks) and A6c134 TCR (yellow sticks) complex structures looking from the side of the MHC groove (gray sticks). **(C)** Comparison of CDR loop conformation in the A6wt TCR (yellow and orange ribbons) and A6c134 TCR (blue and green ribbon) A2-Tax complex structures. **(D)** Crossing angle comparison of the A6wt TCR and A6c134 TCR in complex with A2-Tax. Colors as in C.

In order to determine the structural basis of the high affinity binding for the A6c134 TCR, we solved the A6c134/A2-Tax complex structure to 2.74 Å. Molecular replacement was successful only in space group C121, consistent with the presence of one molecule of the complex per asymmetric unit, and the resolution was sufficiently high to show that the interface between the two molecules was well ordered and contained well defined electron density. The crystallographic R/R_free_ factors were 22 and 26%, within the accepted limits shown in the theoretically expected distribution (Tickle et al., 2000) (Table S1 in Supplementary Material). The overall buried surface area (BSA) of 2445.4 Å (TCR/pMHC) was within the range observed for previously characterized TCR/pMHC interactions (Rudolph et al., 2006). The high affinity A6c134 TCR bound with a diagonal docking geometry to A2-Tax and showed one to one stoichiometry as previously reported of other TCR/pMHC complexes (Rudolph et al., 2006). We observed a high level of similarity between the A6wt/A2-Tax and A6c134/A2-Tax complexes, suggesting that overall conformation was unaffected by the mutations in A6c134. Importantly, the Tax peptide conformation was virtually identical in both complexes, discounting the possibility that structural changes in the peptide contributed to the high affinity observed (Figures 1A,B). Similarly, the architecture of the CDR loops was unaffected by the mutations in the A6c134 TCR (Figure 1C), and the crossing angle of both TCRs was identical at 34° (Figure 1D) and fell within the previously observed range (Rudolph et al., 2006). Thus, differences in binding affinity between the A6wt and A6c134 TCRs could not be explained by a large conformational change in geometry, consistent with observations in similar studies with other systems (Dunn et al., 2006; Sami et al., 2007; Madura et al., 2013).

### The A6c134 TCR CDR loops undergo large conformational adjustments during A2-Tax engagement

**Figure 2:**
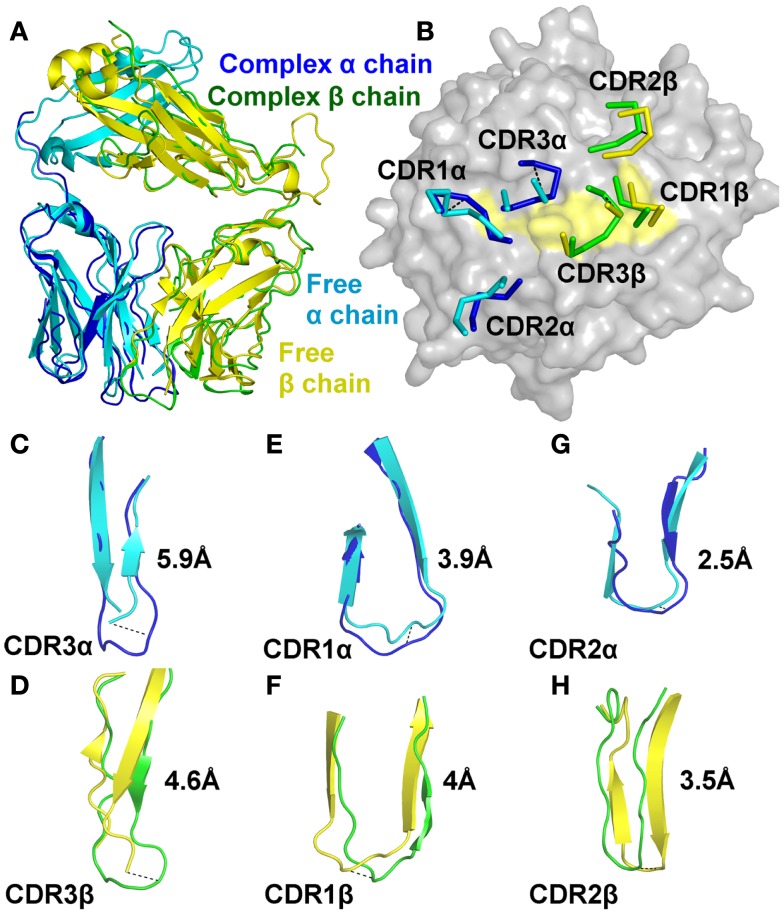
**The A6c134 TCR CDR loops undergo a large degree of conformational adjustment during binding to A2-Tax**. Comparison of the conformations of the A6c134 TCR CDR1, CDR2, and CDR3 loops in the A6c134/A2-Tax complex versus A6c134 TCR free structures. **(A)** Superposition of the free (cyan and yellow cartoon) and complexed (blue and green cartoon) A6c134 TCR. **(B)** Superposition of the free (cyan and yellow lines) and complexed (blue and green lines) A6c134 TCR looking down on the A2-Tax surface (gray and yellow surface). **(C)** Superposition of the free (cyan cartoon) and complexed (blue cartoon) A6c134 TCR CDR3α loop. **(D)** Superposition of the free (yellow cartoon) and complexed (green cartoon) A6c134 TCR CDR3β loop. **(E)** Superposition of the free (cyan cartoon) and complexed (blue cartoon) A6c134 TCR CDR1α loop. **(F)** Superposition of the free (yellow cartoon) and complexed (green cartoon) A6c134TCR CDR1β loop. **(G)** Superposition of the free (cyan cartoon) and complexed (blue cartoon) A6c134 TCR CDR2α loop. **(H)** Superposition of the free (yellow cartoon) and complexed (green cartoon) A6c134TCR CDR2β loop. Loop moments at the apex, or region of greatest movement are shown.

We next solved the structure of the A6c134 TCR at 2 Å (Table S1 in Supplementary Material; Figure 2). The crystallographic R/R_free_ factors were 23.4 and 29.5%, consistent with the expected ratio range (Tickle et al., 2000). In many cases, although not all (Borbulevych et al., 2011b; Holland et al., 2012), TCR CDR loops have been shown to undergo numerous, and sometimes large, conformational changes upon pMHC binding (Armstrong et al., 2008b). Superposition of the free and the bound A6c134 TCR showed that, although the overall conformation of the TCR was virtually identical (Figure 2A), the CDR loops underwent substantial movements (Figure 2B). The CDR3 loops of both chains of the A6c134 TCR were poorly ordered and could not be fully resolved, indicating a large amount flexibility in this region of the TCR. Although the apex of the CDR3 loops could not be accurately located, the portions of the CDR3α and CDR3β loops that were visible underwent large hinge movements of ∼5.9 and ∼4.6 Å, respectively (Figures 2C,D). These changes were substantial compared to other structural studies in which the largest loop movement observed for a human MHCI restricted TCR was 5.6 Å (Kjer-Nielsen et al., 2002; Stewart-Jones et al., 2003). Interestingly, a nearly identical observation was made for A6wt TCR (Scott et al., 2011), for which the dynamics of the CDR3 loops were shown to have a large influence on the specificity and cross-reactivity of the TCR. This occurrence with A6c134 leads to the somewhat counterintuitive conclusion that substitution of the sequence AGGR with MSAQ in CDR3β does not greatly impact the overall dynamics of the loop, and leads us to suggest that the enhanced affinity of A6c134 was not attributable to “preorganization” of CDR3β, i.e., the MSAQ mutation did not alter the non-bound form of the A6c134 TCR to a conformation closer to that of the bound form. This conclusion is consistent with the observation that the binding of A6c134 to A2-Tax is characterized by a much more favorable enthalpy change along with a less favorable entropy change (as pre-organization would have resulted in a more favorable binding entropy) (Armstrong and Baker, 2007; Piepenbrink et al., 2009).

The CDR1 and CDR2 loops underwent a smaller rigid body shift and hinge movements of 2.5–4 Å (Figures 2E–H). On average, the A6c134 TCR CDR loops moved by 4.1 Å. Altogether, this degree of conformational plasticity is high compared to other TCRs (Armstrong et al., 2008b), and demonstrated that the A6c134 TCR undergoes a high degree of conformational melding during binding, as does the A6wt TCR (Borbulevych et al., 2011a). These movements enabled direct contacts between the A6c134 TCR and A2-Tax and resolved steric clashes that would have occurred between the unbound TCR and the MHC surface, as observed for the A6wt TCR (Scott et al., 2011).

### The high affinity binding of the A6c134 TCR was governed by increased peptide contacts

**Figure 3:**
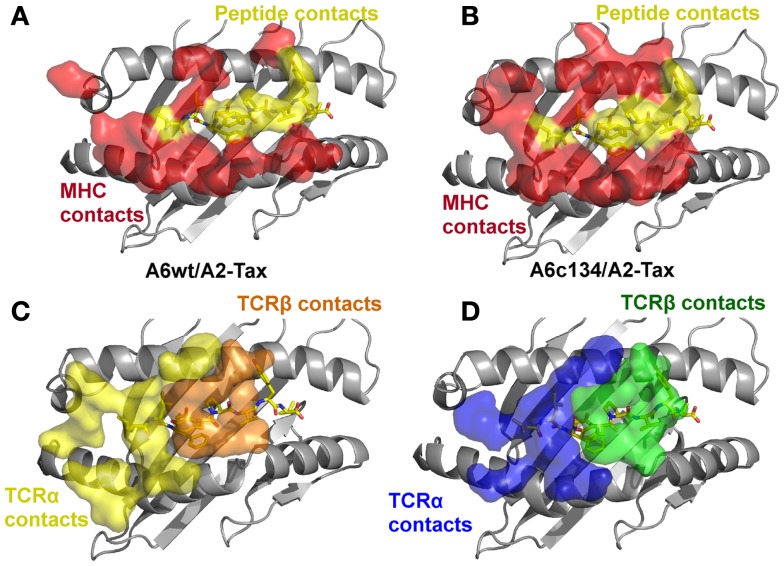
**The specific footprints made in the A6wt TCR/A2-Tax and A6c134 TCR/A2-Tax complex structures are unique**. **(A)** MHC (red surface) and peptide (yellow surface) residues that are contacted by the A6wt TCR. **(B)** MHC (red surface) and peptide (yellow surface) residues that are contacted by the A6c134 TCR. **(C)** A6wt TCR residues (orange and yellow surface) that contact the pMHC. **(D)** A6c134 TCR residues (blue and green surface) that contact the pMHC. Although the overall contact footprint is similar, the A6c134 TCR makes new interactions with both the MHC surface and the peptide.

As the overall free and bound conformations of A6wt and A6c134 were nearly identical, we decided to investigate differences in atomic interactions at the A6wt and A6c134 interfaces. The binding footprints of the A6wt and A6c134 TCRs on A2-Tax were similar, but not identical, resulting in the involvement of different peptide and MHC residues at the interface (Figures 3A,B). Although the A6c134 TCR α-chain contained no mutations, it utilized a different combination of TCR residues for binding A2-Tax compared with A6wt. As a result, the A6c134 TCR α-chain made a number of new and different contacts with A2-Tax compared with the A6wt TCR (Tables S2 and S3 in Supplementary Material; Table 2; Figures 3C,D). Thus, the mutations in the A6c134 TCR β-chain mediated a knock-on, or indirect effect resulting in a modified binding mode for the TCR α-chain.

**Table 2:** **Direct contacts made by the A6wt TCR, or A6c134 TCR CDR3 loops**.

	TCR	vdW (3.2–4 Å)	H-bonds (≤3.4 Å)
A6wt TCR	Thr93	0	1
CDR3α	Thr98	2	1
	Asp99	15	3
	Ser100	7	2
	Trp101	5	0
	Gly102	0	0
	Thr93	1	0
A6c134 TCR	Thr98	2	1
CDR3α	Asp99	15	2
	Ser100	12	2
	Trp101	9	0
	Gly102	3	0
	Arg95	0	1
A6wt TCR	Leu98	14	1
CDR3β	Ala99	0	0
	Gly100	1	0
	Gly101	6	2
	Arg102	14	1
	Pro103	5	0
	Arg95	2	1
A6c134 TCR	Leu98	17	0
CDR3β	Met99	4	0
	Ser100	6	0
	Ala101	17	1
	Gln102	7	0
	Pro103	11	0

*Mutated residues are shown in red*.

**Figure 4:**
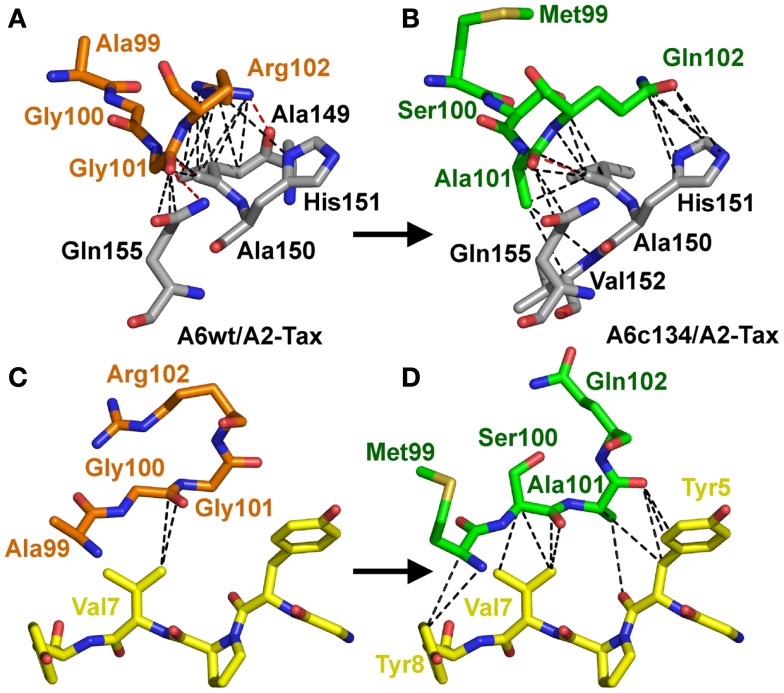
**Increased peptide contacts between A6c134 TCR and A2-Tax mediate the high affinity binding**. Specific contacts (<4 Å) made by residues 99–102 in the TCR CDR3β-chain of either: AGGR (A6wt TCR) or MSAQ (A6c134 TCR). **(A)** Contacts (dotted lines) between the A6wt TCR CDR3β chain, residues 99–102 (AGGR) (shown in orange sticks) and the MHC (gray sticks). **(B)** Contacts (dotted lines) between the A6c134 TCR CDR3β chain, residues 99–102 (MSAQ) (shown in green sticks) and the MHC (gray sticks). **(C)** Contacts (dotted lines) between the A6wt TCR CDR3β chain, residues 99–102 (AGGR) (shown in orange sticks) and the peptide (yellow sticks). **(D)** Contacts (dotted lines) between the A6c134 TCR CDR3β chain, residues 99–102 (MSAQ) (shown in green sticks) and the peptide (yellow sticks). Contacts between the A6c134 and the MHC remain similar to the A6wt/A2-Tax complex whereas A6c134-peptide contacts are increased.

In contrast with the A6c134 TCR α-chain, the A6c134 β-chain containing the mutated MSAQ motif formed a virtually identical footprint on A2-Tax compared to the A6wt TCR (Figure 3D). The A6c134 β-chain made a similar number of contacts with the MHC compared to the corresponding residues in the A6wt TCR (AGGR) (Figures 4A,B). This was reflected by the observation that the A6c134 TCR made only 11 more contacts with the MHC surface compared to the A6wt/A2-Tax complex. However, the A6c134 TCR made 26 extra contacts with the Tax peptide compared to the A6wt TCR, suggesting a TCR-peptide mediated mechanism for the enhanced affinity observed. This observation was also supported by the increase in shape complementarity index (Lawrence and Colman, 1993; Reinherz et al., 1999) (SC = 0.74) for the A6c134/A2-Tax complex compared to the A6wt/A2-Tax complex (SC = 0.63), and is consistent with the observation that the enhanced affinity of A6c134 was enthalpically driven as noted above (Armstrong and Baker, 2007; Piepenbrink et al., 2009).

**Figure 5:**
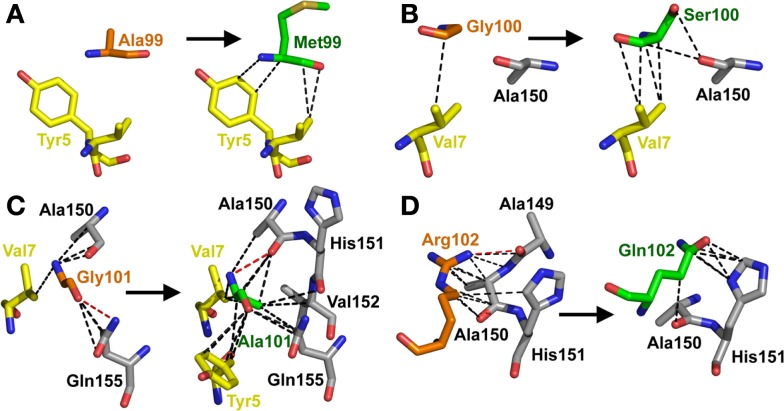
**The MSAQ motif (A6c134 TCR) makes an increased the number of contacts with A2-Tax compared to AGGR (A6wt TCR)**. **(A)** Differences in contacts made by A_99_ (A6wt TCR in orange sticks, left panel) compared to M_99_ (A6c134 TCR in green sticks, right panel). **(B)** Differences in contacts made by G_100_ (A6wt TCR in orange sticks, left panel) compared to S_100_ (A6c134 TCR in green sticks, right panel). **(C)** Differences in contacts made by Gly101 (A6wt TCR in orange sticks, left panel) compared to Ala101 (A6c134 TCR in green sticks, right panel). **(D)** Differences in contacts made by R_102_ (A6wt TCR in orange sticks, left panel) compared to Q_102_ (A6c134 TCR in green sticks, right panel). In all cases, VdW contacts are shown as black dotted lines (4 Å cut-off) and hydrogen bonds/salt bridges are shown as red dotted lines (3.4 Å cut-off). Peptide residues are shown as yellow sticks and MHC residues are shown as gray sticks.

Overall, the mutated MSAQ motif directly accounted for 11 of the 37 new contacts with the surface of A2-Tax (Figures 4C,D; Table 2). For instance; the A_99_–M_99_ mutation generated 4 additional van der Waals contacts (Figure 5A), G_100_–S_100_ generated an additional 5 additional van der Waals contacts (Figure 5B) and G_101_–A_101_ generated 10 additional contacts (Figure 5C), with A2-Tax. Interestingly, the R_102_–Q_102_ mutation resulted in the loss of seven van der Waals contacts and one hydrogen bond (Figure 5D). However, the overall affinity was stronger for A6c134 (MSAQ) compared to A6c134R (MSAR) suggesting that the R_102_–Q_102_ mutation contributed indirectly to binding. Thus, 26 new contacts were generated through indirect interactions with non-mutated residues in the c134 TCR. The majority of these (21 new contacts) were made between non-mutated residues in the c134 TCR CDR3α and CDR3β loops demonstrating that the proximity of residues to the mutated MSAQ motif in the CDR3β loop was probably important for enabling the formation of these new interactions (Table 2).

## Discussion

Thymic selection generates T cells that express TCRs with a weak binding affinity (*K*_D_ = 100 nM–270 μM) for cognate antigen (Cole et al., 2007; Bridgeman et al., 2012). Presumably, this affinity range is important to ensure host protection against foreign invaders, whilst maintaining tolerance to self-antigens from a T cell repertoire of around 25 million (Arstila et al., 1999). In order to perform this function, growing evidence suggests that TCRs must be highly cross-reactive within the confines of MHC-restriction (Mason, 1998; Sewell, 2012; Wooldridge et al., 2012). We have previously shown that it is possible to enhance TCR binding affinity using phage display (Li et al., 2005). These engineered high affinity TCRs represent a potentially useful tool to target specific disease molecules, such as cancer (Liddy et al., 2012) or HIV (Varela-Rohena et al., 2008). However, how enhanced affinity affects TCR binding and specificity is not fully understood, and there are likely multiple mechanistic routes through which affinity can be enhanced. A greater understanding of such mechanisms is particularly important when developing T cell therapies that involve genetically modifying T cells with enhanced affinity TCRs or when using soluble high affinity TCR therapies, to limit potentially dangerous self-reactivity.

In order to explore how TCR CDR3 loop mutations could influence TCR binding and enhance affinity, we generated a modified TCR with nearly 1000-fold enhanced binding affinity by mutating just four residues in the TCR CDR3β loop. Despite the enhanced binding affinity, the A6c134 TCR utilized a native binding mode with diagonal binding geometry as observed for other TCR/pMHC complexes (Rudolph et al., 2006). This observation is similar to other high affinity TCR structures that have been reported previously (Dunn et al., 2006; Sami et al., 2007; Madura et al., 2013). Furthermore, comparing the structures of the free and complexed A6c134 TCR demonstrated that the TCR CDR3 loops underwent a reduction in conformational flexibility upon ligand binding similar to that observed with the A6wt TCR. Thus, despite the mutation of two glycines in the CDR3β loop, high affinity binding did not seem to result from preorganization of the TCR binding site.

We observed that the total number of contacts across the interface was greater for A6c134/A2-Tax than for A6wt/A2-Tax. For instance, in the A6c134/A2-Tax complex, there were a total of 154 contacts, including 72 to peptide and 82 to the MHC. In the A6wt/A2-Tax complex, there were total of 117 contacts, including 46 to peptide and 71 to the MHC. Both the MHC and peptide were involved in generating these new contacts, and they were mediated by both mutated and non-mutated TCR residues. However, the majority of the new contacts arose from interactions between the peptide and the c134 TCR CDR3 loops. Thus, we concluded that higher affinity was mediated predominantly by new TCR-peptide interactions. This conclusion, that new contacts mediated the stronger binding affinity, is consistent with previous thermodynamic analyses, which showed that the A6c134 TCR bound to A2-Tax with a substantially more favorable enthalpy compared to the A6wt TCR (ΔΔH = −10 kcal/mol) (Armstrong et al., 2008a; Piepenbrink et al., 2009).

Our generation of a high affinity TCR that contained mutations at only four residues in the CDR3β loop compared to the wt TCR sequence raises the question of why high affinity TCRs are not naturally selected in the thymus (Holler et al., 2003). Clearly, the structural framework of the TCR allows for high affinity binding, and the mutations we have identified fall within the rearranged gene segment of the TCR rather than the pre-defined germline encoded segments. Furthermore, the mutations in the TCR CDR3β chain generated an increase in peptide contacts within the boundaries of the native A6wt TCR binding mode and would thus be unlikely to alter MHC-restriction. Therefore, it seems very likely that high affinity TCR variants could be generated during the process of TCR rearrangement in the thymus. Yet such high affinity TCRs have not been observed during the peripheral immune response, implying that they are negatively selected (Holler et al., 2003). Presumably, this process is designed to limit self-reactivity. However, weaker affinity TCRs may also be selected to ensure a level of T cell cross-reactivity capable of fully protecting the host against all possible disease epitopes (Mason, 1998; Sewell, 2012; Wooldridge et al., 2012). In support of this notion, our data indicate that A6c134 and other high affinity TCRs can retain extremely high levels of specificity, and may be more specific than their wild-type parents (Laugel et al., 2005; Dunn et al., 2006; Madura et al., 2013). Generally, therapeutic TCRs do not need the capacity to be cross-reactive as they are designed to target a single disease epitope. This difference in desired function (immune response versus specific therapy) may represent an opportunity to improve the affinity of natural TCRs in a safe manner. Thus, our structural investigation of A6c134/A2-Tax, showing that the high affinity interaction was mediated by a native binding mechanism that was peptide-focused, may represent an important approach for developing antigen specific high affinity TCR reagents for use in T cell based therapies.

## Materials and Methods

### Phage display

Selection of high affinity A6wt TCR variants was performed as previously described (Li et al., 2005).

### Protein purification

A2-Tax peptide-MHC complexes was prepared as previously described (Garboczi et al., 1992), by expressing HLA-A^∗^0201 heavy-chain truncated at residue Pro-276 and β2 microglobulin separately in *E. coli* in the form of inclusion bodies, followed by *in vitro* refolding with synthetic peptide. pMHC for binding analysis was prepared similarly, but with the MHC fused to a biotinylation tag (Cull and Schatz, 2000) which was biotinylated *in vitro* by the BirA enzyme (O’callaghan et al., 1999). Disulfide-linked A6c134 TCR was prepared as previously described (Boulter et al., 2003; Li et al., 2005).

### Binding analysis by surface plasmon resonance (Biacore™)

Binding analysis was performed on a Biacore™ 3000 machine using a CM-5 (research grade) chip as previously described (Cole et al., 2008; Miles et al., 2011). Streptavidin was immobilized on all flow cells using amine coupling to a level of>1000 RU (response units). Biotin tagged peptide-MHC was flowed over the streptavidin coated surface at a concentration of approximately 10 μg/ml until ∼150 RU pHLA was bound. Control surfaces were coated with non-cognate pMHCs (A2-ILAKFLHWL, A2-ELAGIGILTV, and A2-YLEPGPVTA) or were left coated with streptavidin. Kinetic binding data were generated using the KINJECT program to inject 10 nM TCR over the flow cells. Data were analyzed using BIAevaluation™ software by kinetic fitting to calculate *k*_on_ and *k*_off_ rates. Binding affinities were calculated using the following equation: *K*_D_ = *k*_off_/*k*_on_.

### Crystallization and X-ray data collection

A6c134/A2-Tax crystals were grown in MES 25 mM pH 6.5, 24% PEG 3350 and 10 mM NaCl; A6c134 free crystals were grown in MES 25 mM pH 6.5, 24% PEG 3350 and 10 mM NaCl. All crystals were soaked in 30% ethylene glycol before cryo-cooling. Data were collected at 100 K at the Advanced Photon Source at Argonne National Laboratory, USA. Reflection intensities were estimated with the XIA2 package (Winter, 2010) and the data were scaled, reduced, and analyzed with SCALA and the CCP4 package (Collaborative Computational Project, Number 4, 1994). Structures were solved with molecular replacement using PHASER (McCoy et al., 2007). Sequences were adjusted with COOT (Emsley and Cowtan, 2004) and the models refined with REFMAC5. Graphical representations were prepared with PYMOL (Delano, 2002). The reflection data and final model coordinates were deposited with the PDB database (A6c134/A2-Tax, PDB: 4FTV; A6c134 free, PDB: 4GRM).

## Conflict of Interest Statement

The authors declare that the research was conducted in the absence of any commercial or financial relationships that could be construed as a potential conflict of interest.

## Supplementary Material

The Supplementary Material for this article can be found online at http://www.frontiersin.org/T_Cell_Biology/10.3389/fimmu.2013.00168/abstract

Supplementary Table S1 **Data collection and refinement statistics (molecular replacement)**.

Supplementary Table S2 **Structural analysis of A6wt/A2-Tax contacts**.

Supplementary Table S3 **Structural analysis for A6c134/A2-Tax contacts (mutant residues in red)**.

## Abbreviations

pMHC, peptide-major histocompatibility complex; RU, response unit; SPR, surface plasmon resonance.
