# Supplementary material for: Increased Peptide Contacts Govern High Affinity Binding of a Modified TCR Whilst Maintaining a Native pMHC Docking Mode
Source: Front Immunol. 2013 Jun 26;4:168. doi: 10.3389/fimmu.2013.00168 (PMC3693486; doi:10.3389/fimmu.2013.00168)
Supplement: Supplementary Table S1 — Data collection and refinement statistics (molecular replacement). [file 54043_Cole_DataSheet2.PDF]

Supplementary data:

Increased peptide contacts govern high affinity binding of a modified TCR whilst maintaining a native pMHC docking mode

David K Cole[1,5*], Malkit Sami[2,5], Daniel R. Scott[3,5] , Pierre J Rizkallah[1], Oleg Y Borbulevych[3], Penio T. Todorov[2], Ruth Karen Moysey[4], Bent K. Jakobsen[2], Jonathan M. Boulter[1$], Brian M Baker[3,5*], Yi Li[2,5]

[1] Institute of infection and Immunity, Henry Wellcome building, Cardiff University School of Medicine, Heath Park, Cardiff, CF14 4XN, United Kingdom.
[2] Immunocore Limited, 57-59 Milton Park, Abingdon, Oxon OX14 4RX, United Kingdom.
[3]Department of Chemistry and Biochemistry, 251 Nieuwland Science Hall, University of Notre Dame, Notre Dame, Indiana 46556
[4] Oxford Nanopore Technologies Ltd., Edmund Cartwright House, 4 Robert Robinson Avenue Oxford Science Park, Oxford, OX4 4GA, United Kingdom
[5] These authors contributed equally to this work
[*] Correspondence: Dr David Cole, Institute of infection and Immunity, Henry Wellcome building, Cardiff University School of Medicine, E-mail: coledk@cf.ac.uk, or Professor Brian Baker, Department of Chemistry and Biochemistry, 251 Nieuwland Science Hall, University of Notre Dame, brian-baker@nd.edu
[$] Deceased 14th May 2008

Keywords:
Human T leukocyte virus type 1 (HTLV-1), crystal structure, peptide-major histocompatibility complex (pMHC), surface plasmon resonance (SPR), T cell, T cell receptor (TCR), A6wt TCR, high affinity TCR.

Non-standard abbreviations:
pMHC, peptide-major histocompatibility complex; RU, response unit; SPR, surface plasmon resonance.

Supplementary Table 1: Data collection and refinement statistics (molecular replacement).

**Data set statistics**	A6c134/A2-Tax	Free A6c134
Space Group	C121	P1 21 1
Unit Cell parameters (Å,°)	a=224.2, b=49, c=94.4	a=90.5, b=52, c=95.2, β=104.4
Radiation Source	APS (19-BM)	APS (19-ID)
Wavelength (Å)	0.985	0.979
Resolution (Å)	2.74	2.00
Unique reflections	26730	57,468
Completeness (%)	100	98.9
Multiplicity	3.6	3.8
I/Sigma(I)	14.9	24
Rmerge (%)	9.5	7
**Refinement statistics**		
No reflections used	26,723	54,513
No reflections in Rfree set	1336	2918
Rcryst (no cutoff) (%)	21.6	23.4
Rfree (%)	26	29.5
Bond lengths (Å)	0.009 (0.021)	0.017 (0.020)
Bond Angles (°)	1.378 (1.938)	1.852 (1.938)
Mean B value ($Å^2$)	34.6	42.9
Outliers Ramachandran plot (%)	0	0
Overall ESU based on Maximum Likelihood (Å)	0.315	0.162
PDB Code	4FTV	4GRM

One crystal was used for data collection.

Supplementary Table 2: Structural analysis of A6wt/A2-Tax contacts

CDR loop	TCR	Peptide	MHC	vdW (3.2 – 4 Å)	H-bonds (≤3.4 Å)
CDR1α	Lys1^{N}		Glu58$^{O\varepsilon2}$	1	1
	Asp26$^{O\delta2}$		Glu58$^{O\varepsilon1}$	6	1
	Arg27		Trp167	3	
	Arg27		Arg170	4	
	Gln30		Lys66	1	
	Gln30		Tyr159	1	
	Gln30		Thr163	1	
	Gln30$^{N\varepsilon2}$	Leu1^{O}		1	1
	Ser31$^{O\gamma}$	Tyr5^{OH}		3	1
CDR2α	Tyr50		Gln155	1	
	Tyr50		Ala158	1	
	Asn52$^{N\delta2}$		Glu166$^{O\varepsilon2}$		1
FWα	Lys68$^{N\zeta}$		Thr163$^{O\gamma1}$		1
	Lys68$^{N\zeta}$		Glu166$^{O\varepsilon1}$	5	1SB
CDR3α	Thr93$^{O\gamma1}$	Tyr5^{OH}			1
	Thr98$^{O\gamma1}$		Arg65^{NH2}	2	1
	Asp99$^{O\delta1/O\delta2}$		Arg65$^{N\varepsilon/N\zeta}$	5	2 H-bonds, 1SB
	Asp99		Lys66	4	
	Asp99	Gly4		4	
	Asp99	Tyr5		2	
	Ser100$^{N/O\gamma}$	Gly4^{O}		2	2
	Ser100	Tyr5		5	
	Trp101		Lys68	1	
	Trp101		Ala69	4	
CDR1β	Glu30$^{O\varepsilon1}$	Tyr8^{OH}		2	1
CDR3β	Arg95^{NH2}	Tyr5^{OH}			1
	Leu98		Gln72	1	
	Leu98	Pro6		2	
	Leu98	Val7		3	
	Leu98^{O}	Tyr8^{N}		8	1
	Gly100	Val7		1	
	Gly101^{N}		Ala150^{O}	2	1
	Gly101^{O}		Gln155$^{N\varepsilon2}$	3	1
	Gly101	Val7		1	
	Arg102^{NH1}		Ala149^{O}	1	1
	Arg102		Ala150	12	
	Arg102		His151	1	
	Pro103		Gln155	1	
	Pro103	Tyr5		4	

Using the previously published A2/A2-Tax structure, PDB: 1AO7 (17)

Supplementary Table 3: Structural analysis for A6c134/A2-Tax contacts (mutant residues in red).

CDR loop	TCR	Peptide	MHC r	vdW (3.2 – 4 Å)	H-bonds (≤3.4 Å)
CDR1α	Arg27^{NH1}		Glu55$^{O\varepsilon1}$		1
	Arg27		Glu58	1	
	Arg27		Tyr59	4	
	Arg27		Trp167	2	
	Gly28		Trp167	2	
	Gly28	Leu1		1	
	Gln30$^{O\varepsilon1}$		Lys66$^{N\zeta}$		1
	Gln30		Tyr159	1	
	Gln30		Thr163	1	
	Gln30	Leu1		1	
	Gln30	Gly4		1	
	Ser31$^{O\gamma}$	Tyr5^{OH}		3	1
CDR2α	Tyr50		Glu154	1	
	Tyr50		Gln155	5	
	Tyr50		Ala158	2	
FWα	Lys68$^{N\zeta}$		Thr163$^{O\gamma1}$	2	1
	Lys68$^{N\zeta}$		Glu166$^{O\varepsilon1}$	4	1SB
CDR3α	Thr93	Tyr5		1	
	Thr98$^{O\gamma1}$		Arg65^{NH2}	2	1
	Asp99$^{O\delta1}$		Arg65$^{N\varepsilon/N\zeta}$	4	1 H-bonds, 1SB
	Asp99		Lys66	4	
	Asp99	Gly4		4	
	Asp99	Tyr5		3	
	Ser100$^{N/O\gamma}$	Gly4^{O}		2	2
	Ser100	Tyr5		9	
	Ser100	Pro6		1	
	Trp101		Arg65	3	
	Trp101		Lys68	1	
	Trp101		Ala69	5	
	Gly102		Arg65	3	
CDR1β	Gln30$^{O\varepsilon1/O\varepsilon2}$	Tyr8^{OH}		3	2
CDR3β	Arg95^{NH2}	Tyr5^{OH}		2	1
	Leu98		Ala69	1	
	Leu98		Gln72	2	
	Leu98		Thr73	1	
	Leu98	Pro6		1	
	Leu98	Val7		4	
	Leu98	Tyr8		8	
	Met99	Val7		2	
	Met99	Tyr8		2	
	Ser100		Ala150	2	
	Ser100	Val7		4	
	Ala101^{N}		Ala150^{O}	4	1
	Ala101		His151	1	
	Ala101		Val152	1	
	Ala101		Gln155	3	
	Ala101	Tyr5		5	
	Ala101	Val7		3	
	Gln102		Ala150	1	
	Gln102		His151	6	
	Pro103		Gln155	5	
	Pro103	Tyr5		6	
